# Action of Molecular Switches in GPCRs - Theoretical and Experimental Studies

**DOI:** 10.2174/092986712799320556

**Published:** 2012-03

**Authors:** B Trzaskowski, D Latek, S Yuan, U Ghoshdastider, A Debinski, S Filipek

**Affiliations:** 1 Faculty of Chemistry, University of Warsaw, ul. Pasteura 1, 02-093 Warsaw, Poland; 2 International Institute of Molecular and Cell Biology, ul. Ks. Trojdena 4, 02-109 Warsaw, Poland

**Keywords:** 3-7 lock, 7TM receptors, allosteric, conserved motifs, dimerization, drug design, G-protein-coupled receptors, GPCRs, ionic lock, membrane receptors, molecular switches, receptor activation, signal transduction, transmission switch, tyrosine toggle switch.

## Abstract

G protein coupled receptors (GPCRs), also called 7TM receptors, form a huge superfamily of membrane proteins that, upon activation by extracellular agonists, pass the signal to the cell interior. Ligands can bind either to extracellular N-terminus and loops (e.g. glutamate receptors) or to the binding site within transmembrane helices (Rhodopsin-like family). They are all activated by agonists although a spontaneous auto-activation of an empty receptor can also be observed. Biochemical and crystallographic methods together with molecular dynamics simulations and other theoretical techniques provided models of the receptor activation based on the action of so-called “molecular switches” buried in the receptor structure. They are changed by agonists but also by inverse agonists evoking an ensemble of activation states leading toward different activation pathways. Switches discovered so far include the ionic lock switch, the 3-7 lock switch, the tyrosine toggle switch linked with the nPxxy motif in TM7, and the transmission switch. The latter one was proposed instead of the tryptophan rotamer toggle switch because no change of the rotamer was observed in structures of activated receptors. The global toggle switch suggested earlier consisting of a vertical rigid motion of TM6, seems also to be implausible based on the recent crystal structures of GPCRs with agonists. Theoretical and experimental methods (crystallography, NMR, specific spectroscopic methods like FRET/BRET but also single-molecule-force-spectroscopy) are currently used to study the effect of ligands on the receptor structure, location of stable structural segments/domains of GPCRs, and to answer the still open question on how ligands are binding: either via ensemble of conformational receptor states or rather via induced fit mechanisms. On the other hand the structural investigations of homo- and heterodimers and higher oligomers revealed the mechanism of allosteric signal transmission and receptor activation that could lead to design highly effective and selective allosteric or ago-allosteric drugs.

## 1 INTRODUCTION

It is estimated that GPCRs are targets for about 50% of drugs currently on the market [1], mainly due to their involvement in signaling pathways related to many diseases i.e. mental [2, 3], metabolic [4] including endocrinological disorders [5, 6], immunological [7] including viral infections [8, 9], cardiovascular [7, 10], inflammatory [11], senses disorders [12] and cancer [13]. The long ago discovered association between GPCRs and many endogenous and exogenous substances, resulting in e.g. analgesia, is another dynamically developing field of the pharmaceutical research [14]. 

With the determination of the first structure of the complex between a G-protein coupled receptor (GPCR) and a G-protein trimer (Gαβγ in 2011 [15, 16] a new chapter of GPCR research was opened for structural investigations of global switches with more than one protein being investigated. The previous breakthroughs involved determination of the crystal structure of the first GPCR, rhodopsin, in 2000 [17] and the crystal structure of the first GPCR with a diffusible ligand (β_2_AR) in 2007 [18]. How the seven transmembrane helices of a GPCR are arranged into a bundle was suspected based on the low-resolution model of frog rhodopsin from cryo-electron microscopy studies of the two-dimensional crystals [19]. The crystal structure of rhodopsin, that came up three years later, was not a surprise apart from the presence of an additional cytoplasmic helix H8 and a precise location of a loop covering retinal binding site. However, it provided a scaffold which was hoped to be a universal template for homology modeling and drug design for other GPCRs – a notion that proved to be too optimistic. Seven years later, the crystallization of β_2_-adrenergic receptor (β_2_AR) with a diffusible ligand brought surprising results because it revealed quite a different shape of the receptor extracellular side than that of rhodopsin. This area is important because it is responsible for the ligand binding and is targeted by many drugs. Moreover, the ligand binding site was much more spacious than in the rhodopsin structure and was open to the exterior. In the other receptors crystallized shortly afterwards the binding side was even more easily accessible to the ligand. New structures complemented with biochemical investigations uncovered mechanisms of action of molecular switches which modulate the structure of the receptor leading to activation states for agonists or to complete or partial inactivation states for inverse agonists. 

In this review we will describe the proposed activation mechanisms together with molecular switches, compare them and try to generalize the findings with respect to the other GPCRs not only from family A (the most populated Rhodopsin-like family) but also other families of these mysterious receptors. The action of molecular switches was most extensively investigated in the case of two types of receptors: rhodopsin and the β-adrenergic receptors. The recent reviews on activation and action of molecular switches in the Rhodopsin family of GPCRs were published in 2009 by Ahuja and Smith [20], Nygaard *et al.* [21] and also by Hofmann *et al.* [22]. Some other reviews on activation mechanisms were published earlier by Strange [23] and recently by Deupi and Standfuss [24]. The two-year period since 2009 has been very fruitful for the GPCR research and provided detailed explanations on how some of the switches work as well as redefined some hypotheses in this field. In a very recent review [25] Unal and Karnik tried to generalize the concept of molecular switches and came to the idea of a coordinated domain coupling in GPCRs which could be a consequence of the dynamic nature of these receptors. According to this hypothesis when a ligand is bound to a receptor extracellular domain a decrease in the intrinsic disorder of this domain cooperatively changes the conformation of the neighboring receptor domain. Certainly, some other original concepts will be emerging based on the still growing number of crystal structures and other data associated with GPCRs and their complexes. 

## 2 SUPERFAMILY OF GPCRs

The superfamily of G-protein coupled receptors (GPCRs) can be divided into five main families: Glutamate, Rhodopsin, Adhesion, Frizzled/Taste2 (consisting of frizzled, smoothened and taste2 receptors), and Secretin, according to the GRAFS classification system [26] which displaced the previous A-F system [27, 28]. The GRAFS system was formed using the Hidden Markov Model approach to analysis of multiple sequence alignments of all GPCRs from 13 eukaryotic genomes. All five families were formed in the early stage of metazoan evolution and the number of GPCRs in each family increased during evolution. At present, sequence diversity of GPCRs and their abundance is enormous, giving organisms more ways to adapt to various environmental conditions [29]. Additionally, the Rhodopsin family, the largest and the best described of all, is divided into four groups: α, β, γ and δ, out of which only the δ group does not have any representative in the PDB database. The above internal classification of the Rhodopsin family is still under discussion as other methods such as NJ (Neighbor-joining) or UPGMA (Unweighted Pair Group Method with Arithmetic mean) provided phylogenetic trees of a different fan-like shape [30, 31]. Lately, using the multidimensional scaling (MDS), a non-phylogenetic statistical method adapted to evolutionary distant sequences, Chabbert and co-workers [32] showed that the Rhodopsin family should be divided into 4 groups. The central group, G0, is formed by peptide receptors, opsins and melatonin receptors. The second group, G1, includes somatostatin and opioid receptors, chemokine and purinergic receptors, proteinase activated receptors and acid receptors. The G2 group is formed by biogenic amine receptors and adenosine receptors. Finally, the G3 group consists of receptors for melanocortin, phospholipids and cannabinoids, glycoprotein hormone receptors and leucine-rich repeat (LRR) containing receptors, prostaglandin receptors and Mas-related receptors. This classification of Rhodopsin GPCRs emphasized the role of proline residues patterns in TM2 (transmembrane helix 2) and TM5 (observed in correlated mutations) which was confirmed by the recently solved CXCR4 structure [33]. 

Despite the large sequence diversity, all GPCRs most probably share the same fold: seven transmembrane helices joined by extracellular and intracellular loops of varied length (see Fig. (**1**)). A 7TM core is well preserved in all known to date protein structures of GPCRs despite the high degree of sequence variability within this region. It is worth noticing that the seven helix bundle is not a unique feature of G-protein coupled receptors, since there are other proteins in eukaryotic and prokaryotic organisms which share this fold. For example in eukaryotes, a 7TM fold appears in high-conductance Ca^2+^-activated potassium channels (BKCa) [34] and in ligand-gated ion channels [35] which do not have any evolutionary relationship with GPCRs. Particularly, in prokaryotes, light-induced structural changes in the 7TM core of bacteriorhodopsin [36, 37] or halorhodopsin [38] provoke pumping of protons (hydrogen ions) or halide ions, respectively, through the membrane, or induce protein-protein interactions which initiate a signaling cascade associated with phototaxis, as in the case of the sensory rhodopsin I-transducer complex [39].

While the 7TM core is a typical common feature of GPCRs, the extracellular and intracellular regions differ in structure, sequence and length allowing interactions with various signaling molecules and ligands: ions, organic odorants, amines, peptides, proteins, lipids, nucleotides and photons [26]. Moreover, many GPCRs, so-called orphan receptors, still lack a reliable assignment of interacting ligands and some of them may not even need ligands for activation but, most probably, are self-activated through heterodimerization [40]. In general, ligands bind to the extracellular loops and the N-terminus, while the intercellular part of GPCRs is involved in protein-protein interactions with G proteins, arrestin or other subunits.

Depending on the GPCR family different regions of receptors are involved in the activation process (see Table **1**). The common role of GPCRs is a signal transmission to the cell interior through interactions with molecules, such as the G protein or arrestin, by changing the structure of their transmembrane domains and/or extracellular and intracellular parts after the ligand binding. G protein-coupled signal transduction involves dissociation of G protein into Gα and Gβγ subunits which modulate enzymes or membrane channels leading to a highly amplified signaling cascade. In the absence of any ligand a G-protein coupled receptor is believed to be in a dynamical equilibrium between the inactive (R) and the less populated active (R*) state. Binding of an agonist molecule (full or partial) is thought to increase the probability of the receptor converting to R* [41]. Such a scheme is preferred in the case of GPCRs with diffusible ligands, however, in the case of rhodopsin, endowed with a very tight ligand binding site, probably the induced fit mechanism is employed [42]. It is possible that in most GPCRs both mechanisms are operational but in different proportions. These two types of activation paths, the dynamic equilibrium of receptor states and the agonist-induced conformational change, will be described in detail later on. Antagonists prevent the binding of both agonists and inverse agonists into the orthosteric site (a binding site for endogenous agonists) but they can also change the receptor structure (or choose a particular state of the receptor according to ensemble of conformational receptor states) which can even induce receptor internalization in some cases. GPCRs activation and signal transmission can also be influenced by allosteric or ago-allosteric modulations induced not only by several ligands known to date [43, 44] but also through either negative or positive cooperation between protomers within a dimer. Many functional studies proved dimerization of GPCRs [45] although even a monomer is able to activate its G protein [46], to bind arrestin or to undergo phosphorylation catalyzed by GRKs (GPCR kinases) [47]. The role of oligomerization in activation and signal transduction by GPCRs is still not clear although some experimental and theoretical studies involving not only Rhodopsin but also the Glutamate and Secretin families proved its relevance [48, 49]. Moreover, many GPCRs, so-called orphan receptors, still lack reliable assignment of interacting ligands and some of them are probably self-activated i.a. through heterodimerization [40]. 

GPCRs were traditionally considered to be monomeric and recent studies of GPCRs reconstituted in high-density lipoprotein particles have confirmed that these receptors can exist and function as monomers [46, 50]. However, as evidenced by biochemical measurements of cooperativity, biophysical determinations of fluorescence or by bioluminescence resonance energy transfer between protomers, co-immunoprecipitation and other methods GPCRs from various families can assemble as dimers or higher-order oligomers [51-53]. Currently, dimerization was proposed to play a role in processes ranging from ligand binding to receptor signaling, maturation, trafficking and regulation. For the glutamate receptor family of GPCRs activation involves a movement of the N-terminal Venus flytrap domain (VFTD) within a dimeric GPCR entity to activate the membranous domains, which suggests that dimerization is mandatory for agonist-induced activation [54]. It is likely that various allosteric interactions between monomers in an oligomeric complex represent those that occur between distinct sites within a given GPCR monomer [55]. To date, experimental data suggest that GPCR dimers and oligomers are functionally asymmetric which was characterized especially for GABA_B_R and the mGluR receptors from the glutamate family [56, 57]. Because of asymmetric functionality the subunits in a GPCR dimer possibly adopt different conformations in a particular receptor state (inactive or active). Recent studies on dopamine D2 receptor dimers have also demonstrated asymmetric communication between an agonist-bound and an antagonist-bound protomers within the D2 dimer [53].

Homo- and hetero-dimerization can modulate the signaling properties of receptors and mediate cross-talk between GPCR pathways [58]. Crystal structures can also directly suggest novel allosteric sites with specific properties and selectivity. For example, a cholesterol binding site located in the interface between protomers consisting of helices TM1 and H8, has been observed in many dimeric β_2_-adrenergic receptor crystal structures [59]. Cholesterol can modulate receptor thermostability and ligand affinities. However, even for the most studied dimers the identification of the functionally relevant interface is still very difficult. This is partly due to the transient mode of the interactions and the technical problems of differentiating between specific and nonspecific binding in membrane environment [60]. On the other hand, it was observed in crystallization studies that nonspecific or partial dimerization of GPCRs can prevent crystal formation because it introduces heterogeneity to the system, so it is usually avoided. Therefore, in most crystal structures of GPCRs analyzed so far the receptor molecules have been found in non-functional (antiparallel or tilting) orientations. The recent crystal structures of CXCR4 [33] are rather exceptions revealing a parallel dimer arrangement involving helices TM5 and TM6. The dimer interface is virtually identical in five different crystal packing forms of CXCR4 with both peptide and small-molecule antagonist, which can suggest that it is functionally relevant [61]).

Using a fluorescence resonance energy transfer (FRET) to characterize the oligomerization of β_2_AR Fung *et al*. [52] proposed that β_2_AR forms predominantly tetramers when reconstituted in phospholipid vesicles. Agonists and antagonists had little effect on the relative orientation of protomers in the oligomeric complexes so it was suggested that the tetramer structure is loose enough to accommodate a large, outward movement of the cytoplasmic part of TM6. In contrast, binding of inverse agonists led to a significant increase in FRET efficiencies for most labeled amino acid pairs. This could suggest that inverse agonists can induce tighter packing of protomers and/or stimulate the formation of larger oligomers (possibly octamers or larger structures) by employing the additional interface at the receptor surface. The results provide new insights into β_2_AR oligomerization and reveal a possible mechanism for the functional effects of inverse agonists. The interface involving helices TM1 and H8 was proposed for a symmetrical dimer (as it was found in the crystal structures of this receptor type) so the tetramer would be a dimer of dimers. Upon binding of an inverse agonist the dimers could form a tighter structure and additionally the tetramers could stick together to form octamer engaging an interface involving helices TM4 and TM5. In this way a larger oligomer can be formed using more tetramers. Similar interfaces were proposed for rhodopsin oligomers based on the AFM measurement [62, 63], however in this case the interface in a rhodopsin dimer involved helices TM4 and TM5 while contacts between rows of dimers were maintained by helices TM1 from different protomers. 

## 3 MECHANISMS OF MOLECULAR ACTIVATION

The idea of a common molecular activation mechanism in G protein-coupled receptors (GPCRs) has been developed in the late 80's based on the structural homology of GPCRs. Despite the wealth of different classes of molecules activating GPCRs it was believed that the activation of all GPCRs utilizes a similar mechanism which triggers the response through a common set of G proteins. Several experiments from the early 90's showed, however, that there is probably no common "lock" for all the members of the GPCR family [64-66]. Molecular switches can be precisely observed in the already solved structures of GPCRs (Fig. (**2**)). However, to search for such strong inter-residue interactions which stabilize either the active or inactive states in GPCRs of unknown 3D structure some sophisticated experimental or theoretical methods must be used. Molecular switches are usually highly conserved (e.g. in the Rhodopsin family). Consequently, multiple sequence alignments and homology modeling combined with molecular dynamics (MD) simulations are often used to capture novel stabilizing interactions especially in GPCRs families other than Rhodopsin.

### 3.1 Glutamate Receptors

The glutamate receptors (previously: class C of GPCRs) are activated through binding of a ligand by a well-conserved so-called “Venus flytrap module” (VFTM) in the N-terminal region consisting of two lobes [67]. Structural changes induced by agonist binding (the closure of lobes) are propagated from the VFTM domain through conserved disulphide bridges within the extracellular cysteine-rich domain (CRD) to the TM core (see Table **1**) [68]. GABA-binding GPCRs from the Glutamate family share also an additional common domain in the N-terminal region: SUSHI (also known as complement control protein modules or short consensus repeats) [69]. Studies on mGlu receptors showed that the simultaneous closure of lobes in the VFTM domain of both protomers in a dimer during activation by an agonist is necessary to maintain the high binding affinity towards the G protein [48]. This suggests that dimerization is a required step for the full performance of Glutamate GPCRs. Glutamate receptors are targets not only for endogenous orthosteric ligands but also for many specific allosteric modulators found to date that interact with TM3, TM5, TM6 and TM7 which are more diverse in sequence than the well-conserved N-terminal region [70]. Due to this considerable sequence diversity in the TM region allosteric ligands, PAMs (positive allosteric modulators) or NAMs (negative allosteric modulators), can be more selective than the orthosteric ones. Most importantly though, HTS (High-Throughput Screening) and the hit optimization of these allosteric ligands combined with the Rhodopsin-based homology modeling and mutagenesis, provided important insights into molecular switches present in Glutamate GPCRs [71], which would not otherwise be possible because of the lack of any representative 3D structure of this receptor family. It was shown that the conformational movement of TM6 with respect to TM3 during the activation process in the mGlu5 receptor could be inhibited by MPEP (NAM) which interacts with aromatic residues in TM3 and TM6 [72, 73] and Ser and Ala in TM7 [74]. Quite often, switching from the PAM to NAM ligands targeting mGlu receptors can be done with very subtle changes of a ligand chemotype, e.g. replacement of fluorine with chlorine atom or fluorine with methyl group (e.g. in analogs of ADX47273) [71]. Such changes would not be of much consequence in the case of orthosteric ligands. The sequence analysis [69] pointed to some weakly conserved motifs in TM6 and TM7 of Glutamate GPCRs (see Table **1**) which could be involved in activation, i.e. ‘wl’ aligning with the Rhodopsin family CwxP motif and ‘pkxy’ which could act similarly to ‘nPxxy’ in the Rhodopsin-like receptors. Uppercase letters in the motifs indicate completely conserved positions, lowercase indicate well-conserved positions (>50%) and x indicates any amino acid.

### 3.2 Adhesion Receptors

The Adhesion family (previousely: class B), for which endogenous ligands have not been identified so far, lacks a complete description of the common activation process. Based on the recent findings on the GPR56 protein, however, it is suggested that a G-protein is involved in signal transduction by this family of receptors [75]. Although conserved polar and aliphatic residues which could be involved in the activation mechanism are present in helices TM3, TM4 and TM6 (see Table **1**) the typical signature of Adhesion GPCRs are highly O- and S-glycosylated long N-termini with the cxCxhlt/s motif inside the GPS domain involved in an autocatalytic proteolytic process. The sequence diversity of the Adhesion family is significant, particularly in the N-terminal region, although some cysteine residues are well-conserved also in the TM core.

### 3.3 Secretin Receptors

A long cysteine-rich N-terminal region with a ligand-binding site (HBD - a hormone-binding domain) is also a typical feature of the Secretin family (previously: class B) but without the presence of a proteolytic domain like in Adhesion receptors. The common motifs including cysteins located in N-termini are CnxxwDxxxxCW and rxCxxxGxw (see Table **1**). In this review we decided to use the following nomenclature [76]: uppercase letters in the motifs indicate completely conserved positions, lowercase indicate well-conserved positions (>50%) and x indicates any, non-conserved amino acid. The active conformation of the receptor is stabilized by simultaneous interactions of the ligand with the N-terminus, extracellular loops and TM6 [77, 78]. Neumann *et al.* proposed that the motif crucial for class B receptors activation is located in the N-terminal helix cap where it interacts with the peptide ligand which forms a well-defined helix upon binding to a receptor [79].

### 3.4 Frizzled Receptors

The most conserved among all GPCRs [29] Frizzled-like receptors contain the N-terminal cystein-bridged ligand-binding region which is involved in the interaction with Wnt glycoproteins. Smoothened receptors belonging to the same family transmit signals through G proteins in a ligand-independent manner as a part of the sonic hedgehog-smoothened complex [80]. In both the Frizzzled and Smoothened receptors conserved cysteine bridges between extracellular loops are indispensable for the proper functioning of receptors but in Taste2 receptors, belonging to the same family, different crucial residues are present in EC1 and EC2 loops, i.a. the NxWaVtnH motif in EC1 loop (see Table **1**) [81]. Recently, Xenopus Fzd3 (Frizzled-like) was found to be functional as a homodimer in transduction of the Wnt/β-catenin signal [82].

## 4 EXPERIMENTAL METHODS TO PROBE ACTIVATION OF RHODOPSIN FAMILY OF GPCRs

### 4.1 Crystal Structures of Rhodopsin and Opsin

The development of crystallographic and spectroscopic techniques in recent years allowed researchers to study in details the activation mechanism of GPCRs. In this section we will describe advances in the techniques and their relevance for investigations of GPCR activation while in Table **2** we provide information about all available crystal structures of GPCRs including the type of a ligand, its name, PDB id, resolution and references to the literature. In 2004 two new structures of bovine rhodopsin were published. First, Okada *et al.* used new crystallization conditions to obtain a full structure of the protein, with no gaps present, at a very accurate, 2.2 Å resolution [83]. Such a good accuracy gave an insight into a chromophore structure, showing e.g. the expected configuration about the C_6_-C_7_ single bond and a twist around the C_11_-C_12_ double bond. In another work Li *et al.* solved the structure of bovine rhodopsin in a trigonal, rather than tetragonal, crystal form [84]. It was interesting to note that even though the packing of rhodopsin molecules in crystals was different, only very minor differences in atom coordinates were present. On the other hand the latter structure helped to solve the positions of all the remaining internal water molecules, which were further stabilizing rhodopsin structure *via *formation of extensive hydrogen-bond networks. Other similar studies confirmed the overall structure of rhodopsin [85, 86]. These studies, while providing a milestone for GPCR structure understanding, gave a rather poor insight into the activation mechanism of GPCRs since in all of them rhodopsin was presumed to be in an inactive state. 

In 2006, however, three different groups performed experiments aiming at elucidation of the rhodopsin structure in different states. First, Nakamichi and Okada studied the differences between rhodopsin and bathorhodopsin using X-ray crystallography under cryogenic conditions with and without illumination [87]. They found some differences in electron densities for these two states, which concerned mostly the retinal molecule, but were relatively small for the residues interacting with it. The same authors managed also to trap rhodopsin in the lumi phase, by illuminating and then successively warming the bathorhodopsin system [87]. In this case they were able to notice a nearly complete all-*trans* conformation of retinal as well as much larger differences in orientations of side chains of the selected residues. They were also able to observe small distortions of the TM3 backbone and changes in the interactions of this helix with TM2 and TM6. Photoactivated rhodopsin was also studied in the work of Salom *et al.* but the resulting crystals were obtained only at the 4.2 Å resolution [88]. Due to low accuracy the authors were able to spot differences between this structure and the ground-state rhodopsin only in portions of the cytoplasmic loops and in the position of retinal in the binding pocket. Both groups concluded that the changes in electron densities between rhodopsin and lumirhodopsin were not as large as expected, particularly for TM6.

A true insight into the active state of rhodopsin became possible in 2008, after Park *et al.* determined the crystal structure of opsin, the first ligand-free form of a GPCR [89]. In general, ligand-free GPCRs are extremely difficult to purify, and in the case of opsin it was known that it is unstable and tends to aggregate. In this case solubilized opsin was crystallized by hanging-drop vapor diffusion, and the obtained crystals were cryoprotected and frozen in liquid nitrogen for X-ray analysis. The comparison between opsin and rhodopsin structures revealed that the TM1-TM4 core remains stable upon activation. On the other hand large changes were observed for the TM5-TM7 region, helix H8 and cytoplasmic loops IC2 and IC3. TM5 is about two helical turns longer than in rhodopsin and tilted in such a way that its cytoplasmic end shifts towards TM6. The cytoplasmic end of TM6 is tilted outward from the helix bundle and closer to TM5. The Arg3.50–Glu6.30 ionic lock is broken as was expected, but other interactions are formed (Arg3.50 interacts with residues on TM5, while Glu6.30 forms hydrogen bonds with residues on TM5 and TM6, see Fig. (**2G**)). The residues are numbered according to the Ballesteros-Weinstein numbering scheme: every amino acid identifier starts with the helix number, followed by the position relative to a reference residue being the most conserved amino acid in that helix which bears number 50 [90]. The cytoplasmic end of TM7 is also rearranged by Tyr7.53 rotation to a position where it blocks TM6 from moving back towards TM3. Finally, in contrast to rhodopsin, the opsin structure shows two openings of the retinal-binding pocket: one between TM5 and TM6 and another between TM1 and TM7. This suggests different retinal entrance and exit routes during receptor activation. These findings were confirmed later in similar study by Scheerer [91].

Advances in crystallization and high-resolution X-ray techniques, which allowed researchers to study the details of rhodopsin system, could not unfortunately be easily transferred to other GPCRs. It has been observed that other membrane receptors, particularly those for diffusible hormones and neurotransmitters (e.g. adrenergic receptors) exhibit significant basal, agonist-independent G protein activation [92, 93]. This basal activity has been associated with structural instability, suggesting that intramolecular interactions maintaining the receptor in the inactive state are also important for the structural integrity of these proteins [94, 95]. This results in difficulties in generating high-quality crystals of many GPCRs, for which alternative methods and techniques are needed. 

### 4.2 Structures of other GPCRs – Stabilization *via *T4L

In 2007 Rasmussen *et al.* obtained a high-resolution (3.4 Å) crystal structure of β_2_AR using a new approach which included generation of a monoclonal antibody (Mab5) that binds to the third intracellular loop of the receptor [96]. Binding of this antibody does not alter agonist or antagonists binding affinities, therefore it was concluded that it should not significantly alter the structure of β_2_AR. The Mab5-receptor complex with bound inverse agonist (carazolol) was stable enough to generate crystals of good quality. In the same year Cherezov *et al.* suggested a different modification in which β_2_AR was modified by inserting T4 lysozyme (T4L) in place of the third intracellular loop [18]. The engineered β_2_AR-T4L complex, again with carazolol, yielded a very stable structure that was solved and refined at 2.4 Å resolution. It was shown that the fused T4L is tilted away from the receptor and its interactions with the protein are minimal (only two salt bridges). It was also shown that there were no significant differences between this structure and the β_2_AR-Mab5 structure studied earlier, although the cytoplasmic end of TM6 was pulled outward in the former structure due to the interactions with T4L. Interestingly, both structures have the Arg3.50-Glu6.30 ionic lock broken, even though the residues that should be forming it are relatively close to each other (Fig. (**2H**)).

The same T4L fusion technique has been used in the recent years to obtain more GPCR structures. In 2008 Hanson *et al.* used the same β_2_AR-T4L chimera to identify a specific cholesterol binding site within this receptor [59], while Wacker showed the binding modes of β_2_AR with antagonists and inverse agonists [97]. In the same year Jaakola *et al.* managed to obtain high-quality crystals (solved at 2.6 Å) of the human A_2A_ adenosine receptor – T4L chimera bound to the ZM241385 antagonist [98]. Similarly to the β_2_AR case, this receptor does not have the ionic lock closed (Fig. (**2I**)). Instead, Asp3.49 forms a hydrogen bond with Tyr3.60 in the IC2 loop while Arg3.50 interacts with Thr2.39 in TM2 additionally restraining the helical conformation of IC2. It was suggested that in the case of the A_2A_ adenosine receptor Arg3.50 may play a role in stabilizing the deprotonated state of the adjacent Asp3.49 residue, which would strengthen the polar interaction between the (d/e)Ry motif, IC2 and TM2 with direct implications on G protein activation. It is also interesting to note that the antagonist is interacting with Trp6.48, even though it is bound in a completely different orientation than retinal in rhodopsin (along the main axis of the receptor, Fig. (**2C**)). 

The lysozyme-stabilization method has also been used recently to obtain crystal structures of the CXCR4 chemokine receptor [33], dopamine D_3_ receptor [99], histamine H_1_ receptor [100] with antagonists as well as the agonist-bound A_2A_ adenosine receptor [101]. Of those structures, only the antagonist-D_3_R complex has the Arg3.50-Glu6.30 ionic lock not broken (analogous to Fig. (**2G**)). On the other hand the crystal structures demonstrated that even when the agonist is covalently bound to the receptor, the FAUC50-β_2_AR-T4L crystallizes in an inactive conformation – the ionic lock is broken but there is no movement of TM6 [102]. There were concerns whether lysozyme fusion alters the pharmacology and overall structure of GPCRs [18]. To answer this question an alternative approach for obtaining GPCR structures has been proposed. This method, called thermostabilization, involved introduction of a small number of point mutations into the receptor, which increase its thermostability and alter the equilibrium between agonists and antagonists conformations.

### 4.3 Thermostabilization by Point Mutations

The first success of this method came in 2008 when Warne *et al.* obtained a 2.8 Å resolution structure of β_1_AR [103]. This GPCR was even more challenging than the previously-crystallized membrane proteins, due to its spontaneous cycling between inactive and active states and overall low stability. Two thermostabilizing mutations, Cys116Leu and Cys358Ala, were introduced to the protein to alter its equilibrium so that the mutant receptor remains in the antagonist state. The resulting construct bound to an antagonist (cyanopindolol) gave high-quality crystals solved at the 2.7 Å resolution. In this structure the Arg3.50–Glu6.30 ionic lock is broken, and instead Asp3.49, Arg3.50 and Tyr3.51 are involved in interactions with intracellular loop 2 (similarly to the structure of adenosine A_2A_R with antagonist [98]). The same group also obtained crystal structures of the same construct bound to four different agonists: R-isoprenaline, R,R-carmoterol, R-salbutamol and R-dobutamine [104]. Interestingly, the overall structures of all agonist-bound systems were very similar to the antagonist-bound one and none of the structures showed the expected movement of TM6 which was observed during photoactivation of rhodopsin. The major differences were noticed in the binding pocket, where Ser5.43 and Ser5.46 had different rotamer conformations than in the antagonist-bound form. Authors concluded that agonist-bound crystal structures represent an inactive state of the receptor formed during initial agonist binding.

The thermostabilization method gave, however, even more insight into GPCR activation when applied to the A_2A_ adenosine receptor. First, Lebon *et al.* used four point mutations (L48A, A54L, T65A and Q89A) to obtain a stable receptor bound to two different agonists, adenosine and NECA [105]. Both ligands bind to the receptor in a similar fashion, which is also close to that shown earlier for the antagonist binding to the A_2A_-T4L chimera. The relative similarity of the whole structure to the A_2A_-T4L structure suggests that it is only a partially activated state of the receptor. The largest deviations from the antagonist-bound structure have been found for TM3 (shifted by 2 Å) and in the conformation of the cytoplasmic ends of helices TM5-TM7. The extent of these changes was, however, much lower than the changes occurring upon rhodopsin activation to opsin. Recent crystal structures of the A_2A_ receptor, which have been obtained both in agonist-bound [98, 105] and antagonist-bound states [106, 107], allowed the GPCR community to obtain another interesting clue concerning receptor activation. Similarly to the β_2_AR case, ligand binding causes small conformational changes within the ligand cavity which are different for different types of ligands. These changes, which include the already known TM6 and TM7 tilting and TM5 movement, change the intracellular interface of the receptor which facilitates binding of G proteins. The Arg3.50-Glu6.30 ionic lock is present in one of the antagonist-bound structures, but broken in the agonist-bound systems (Fig. (**2**)).

Recently, Dore *et al.* have used the same technique to obtain A_2A_ adenosine receptor crystals bound to three different antagonists, XAC, caffeine and ZM241385 [106]. In this case, however, a different set of stabilizing mutations (A54L, T88A, K122A, V239A, R107A, L202A, L235A, S277A) was chosen. The latter structure (A_2A_R bound to ZM241385) is particularly interesting, since it allows one to compare exactly the same system obtained using two different methods, thermostabilization and T4L-fusion. The structures are, as expected, similar, raising confidence that both methods provide an effective approach to GPCR crystallization and do not result in major structural abnormalities. There is still a small chance that crystallization itself may introduce some unnatural structural features, but a good agreement between GPCR structures and their chemistry makes it rather unlikely. There are, however, some differences in TM5 and TM6, which are interesting from the structural point of view. TM5 has its C-terminal part moved away from TM6, with respect to the A_2A_R-T4L structure, and TM6 has rotated away from TM5. This results in the presence of the Arg3.50-Glu6.30 ionic lock, which is in a similar conformation as in the rhodopsin structure. Some additional differences in the binding pocket suggest that A_2A_R structures with bound ZM241385, obtained using different crystallization methods, show, in fact, the protein in different conformational states. On the other hand, it was shown that the effect of T4L fusion is specific, since in the D_3_R-T4L structure the ionic lock was present.

### 4.4 Nanobody and G Protein Facilitate Agonist Binding

Recently, the crystal structure of a GPCR has also been obtained using a slightly different crystallization approach. In 2011 Rasmussen *et al.* used a recombinant minimal-sized intact antigen-binding domain of a camelid heavy chain antibody, called nanobody, to stabilize the β_2_AR-T4L structure bound to the BI-167107 agonist [108]. It was found that the nanobody exhibits a G protein-like behavior, promoting activation of the GPCR. The resulting, 3.5 Å structure, represents the protein in the active state, with TM5 and TM6 displaced and TM3 and TM7 moved inward with respect to the inactive β_2_AR-T4L-carazolol structure. The largest change upon activation occurs at TM6, which shows a 11.4 Å movement of Glu6.30, caused by a clockwise rotation of TM6 close to the conserved Pro6.50. Also, the salt bridge between Asp3.49 and Arg3.50 is broken. These changes are almost identical to the changes caused by rhodopsin activation to opsin. On the other hand, the changes in the binding site of β_2_AR upon activation are rather subtle and centered around Ser5.46 in TM5. These changes are, however, propagated to other parts of the protein, rearranging the hydrogen bond network and, in turn, causing relatively large movements and rotations of helices.

Very recently a 3.2 Å structure of the β_2_AR complex with a heterotrimeric GTP binding protein (Gs), a first high-resolution view of transmembrane signaling by a GPCR, was obtained [96]. A stable β_2_AR-Gs system was prepared by mixing the purified GDP-Gs with a molar excess of β_2_AR, stabilized by both T4L and a new nanobody, and bound to the BI-167107 agonist. Later, GDP released from Gs was hydrolyzed and the complex was purified by different types of chromatographies. The largest difference between the G protein-coupled structure and the carazolol-bound inactive structure of β_2_AR is the 14 Å outward movement o TM6 and a smaller outward movement of TM5. Overall, this structure is very similar to the active β_2_AR-T4L-nanobody structure described earlier, with the root mean square distance of approximately 0.6 Å. This result gives confidence that both experimental structures are correct and this method does not introduce any significant errors. 

### 4.5 Probing Stability by Mechanical Unfolding

Activation of GPCRs which operates *via *the action of molecular switches involves conformational changes of the protein. However, different parts of these receptors resist the motion in different ways depending on the number and the quality of interactions stabilizing particular protein fragments. Using AFM (Atomic Force Microscopy) and especially its variations: SMFS (Single Molecule Force Spectroscopy) and DFS (Dynamic Force Spectroscopy), it is possible to estimate the rigidity and other mechanical and kinetic parameters of stable structural segments. Both methods involve mechanical unfolding of the investigated proteins. The experiment starts with an attachment of a protein to a tip of a cantilever which is then retracted perpendicularly to the surface of a membrane bilayer. During unfolding the forces needed to break the interactions stabilizing the protein are recorded together with the elongation of the backbone. In SMFS the unfolding is performed with a constant loading rate (a product of speed and a spring constant) while the DFS method consists of a series of SMFS experiments over a broad range of loading rates.

Up to now the mechanical unfolding experiments were performed on rhodopsin - the only GPCR with the entire structure known. Analysis of DFS results of pulling of rhodopsin starting from the N-terminus led to the determination of positions of the stable structural segments [109]. It was revealed that almost all such segments contain secondary structure elements: segment 1^st^ and 2^nd ^– N-terminus; 3^rd^ – TM1-IC1-TM2; 4^th^ – loop EC1; 5^th^ – TM3-IC2-TM4-EC2; 6^th^ – TM5-IC3-part of TM6; 7^th^ – second part of TM6-EC3-TM7; 8^th^ – H8; 9^th^ – C-terminus. Only the N-terminus and TM6 were divided into two separate segments. In the case of rhodopsin which lacks the disulfide bond Cys110-Cys187 a division into segments differs from that of wild type rhodopsin starting from the 5^th^ segment, as this covalent bond keeps two helixes and two loops tightly together [110]. A comparison of the determined segments with the most conserved residues within GPCRs showed that those residues (including highly conserved motifs (d/e)Ry and nPxxy among others) are placed inside the stable structural segments. Because their positions are less likely to be altered during small conformational changes they certainly favor the creation of key interactions crucial for the functionality of rhodopsin. The DFS experiment revealed that segments 5^th^ and 6^th^ have the highest rigidity compared to the rest of the rhodopsin segments. The mixed composition of the rigid and flexible segments around the retinal binding cavity can be explained by two roles they perform: maintaining inactive receptor state but also allowing for conformational changes during activation [111]. Additionally, the unfolding profiles (force-displacement F-D curves) and positions of stable segments of bovine and mouse rhodopsin proved to be very similar in spite of 23 different residues in their sequences. This may indicate that the network of molecular interactions stabilizing the inactive structure and the functional state (receptor switches) is largely conserved [109]. To reveal how proteins are unfolded some theoretical calculations were also performed by Fanelli and Seeber [112] who investigated the influence of single point mutations on stability of rhodopsin. They applied Steered Molecular Dynamics (SMD) simulations to screen through 20 mutants linked to the autosomal dominant form of retinitis pigmentosa. Unfolding curves exhibited three force peaks, similarly to previous SMFS experiments but without details characteristic to those experiments, therefore, no significant differences in the unfolding pathways for different mutants were obtained. However, the unfolding speed in these simulations was about eight orders of magnitude higher than the experimental one so, possibly, similar investigations using a much lower unfolding speed could reproduce details of the F-D curves and reveal unfolding events as well as the role of molecular switches in keeping the structural segments of rhodopsin highly stable.

## 5 SWITCHES IN RHODOPSIN-LIKE RECEPTORS 

The activation of GPCRs occurs most probably through series of conformational changes called molecular switches. The crystal structures enables the researchers to almost see them in action by comparing structures of the same receptors with agonists and antagonists (Fig. (**2**)). Based on the crystal structures we describe those molecular switches that are well characterized and proposed to work in most of GPCRs. 

### 5.1 The Ionic Lock Switch

The presence of the first switch, the ionic lock, has been shown in the first GPCR X-ray structure obtained by Palczewski *et al.* [17]. The inactive state of bovine rhodopsin shows a strong intramolecular interaction between residues Glu3.49/Arg3.50 of the conserved (d/e)Ry motif in TM3 and residues Glu6.30/Thr6.34 in TM6 (Fig. (**2G**)). The authors of that paper concluded that "it could be one of the critical constraints keeping rhodopsin in the inactive occupation", but also noted that this region has high crystallographic B-values, meaning that the side chains may assume different conformations. Following that work as well as mutagenesis studies, which showed the importance of the (d/e)Ry motif [113], the activation mechanism of GPCRs has been described as a cascade of altering molecular switches in conserved microdomains [20, 114]. In this mechanism, ligand binding triggers a series of molecular switches (including the TM3-TM6 ionic lock) to unlock the G protein-binding site in the intracellular face of the receptor, leading to G protein activation.

Apart from inactive rhodopsin there are only few crystal structures in which this particular ionic lock is observed. The dopamine D_3_ receptor [99] and adenosine A_2A_ receptor [106] (but only with selected antagonists) are the only other GPCRs that show the Arg3.50-Glu6.30 ionic lock in the crystal structure. In addition, the residues Asp3.49 and Arg3.50 are forming hydrogen bonds with Tyr3.60 (located in IC2), possibly stabilizing the ionic lock and restraining a helical conformation of IC2. The turkey β_1_-AR structure has the ionic lock open but because of the close proximity of helices TM3 and TM6, the different rotamers of these residues would yield the switch closed. The A_A2_R-T4L chimera has a similar structure in this region and the (d/e)Ry motif is forming a Asp3.49-Tyr3.60 hydrogen bond which restrains the conformation of intracellular loop 2 (IC2). In the human β_2_AR structure the ionic lock between Arg3.50 and Glu6.30 is absent; instead, a hydrogen bond between the highly conserved Tyr3.60 and His6.31 is present. CXCR4 is lacking the Glu6.30 residue and no ionic lock is present between TM3 and TM6. In the histamine H_1_ receptor the ionic lock is also absent; instead, Arg3.50 adopts a different conformer and forms a hydrogen bond with Gln6.36 in TM6 which can also bridge helices TM3 and TM6 to some extent. The lack of the ionic lock despite the presence of residues capable of forming strong interactions has been intriguing; some attribute it to the inclusion of the T4L fusion protein in crystal structures, which may affect the interactions in TM6.

### 5.2 The 3-7 Lock Switch

In rhodopsin the key restrain which is broken first upon retinal isomerization is a salt bridge between a protonated Schiff-base of retinal-Lys7.43 and a counterion, Glu3.28 (Fig. (**2J**)). This switch is called the 3-7 lock because a link between TM3 and TM7 is broken during activation. A similar mechanism probably exists in other receptors, especially with amine-type ligands (aminergic receptors) e.g. histamine H_1_ [100] or dopamine D_3_ [99], which were crystallized with antagonists bound and also in opioid receptors (OPR) for which an extensive modeling was done [115-120]. In these receptors the switch is composed of different residues: Tyr7.43 (which is more conserved than lysine present in rhodopsin) and Asp3.32 which substitutes for the rhodopsin’s counterion, Glu3.28. Asp3.32 is located on the same face of TM3 and deeper in the receptor interior which compensates for a shorter length of its side chain. In β_1_- and β_2_-adrenergic receptors there is also a hydrogen bond, Asp3.32-Tyr7.43, but additionally, the Asn7.39 residue, positioned one turn of helix away of Tyr7.43, is linked to Asp3.32 by a protonated nitrogen atom of aminergic receptor ligands. This is why disruption of the Asp3.32-Tyr7.43 hydrogen bond does not break the link between TM3 and TM7 so the 3-7 lock switch is not functioning in adrenergic receptors. Opening of the 3-7 lock was suggested by Khorana [121] to be the first switch activated in rhodopsin and possibly it is one of the first switches that can be activated upon ligand binding in some other GPCRs. In Fig. (**2**) it is represented by one panel with rhodopsin structures. 

### 5.3 Transmission Switch (Former Trp Rotamer Toggle Switch)

In all crystal structures with agonists there are movements of TM5 and TM6 but they vary considerably. Several similarities can be observed including a relocation of conserved residues Trp6.48 and Phe6.44 towards Pro5.50 (Fig. (**2A-C**)). Such movements were called “a transmission switch” by Deupi and Standfuss [24] instead of the previous name “rotamer toggle switch”. This novel and larger switch links the agonist binding site with the movement of TM5 and TM6 through rearrangement of the TM3-5-6 interface. This is possibly the most common switch among GPCRs. After movement of Trp6.48 in rhodopsin the Phe6.44 residue situated one helix turn away toward the cytoplasmic side of TM6, is displaced toward Leu5.51 as the whole TM6 is rotating horizontally. In β-adrenergic receptors a little contraction of the binding site is observed while in rhodopsin isomerization of retinal makes the binding site much larger. The interaction of Ser5.42 and Ser5.46 with agonists stabilizes the receptor conformation which leads to a 2.1 Å movement of TM5 and about a 1.4 Å movement of Pro5.50 whereas, unlike in rhodopsin, there is no movement of Trp6.48. However, there is a rotation and movement but only of the cytoplasmic part of TM6. This is translated to a relocation of Ile3.40 from its position at Pro5.50 and a motion of Phe6.44 due to rotation of TM6. Activation of this switch seems to be limited to some classes of GPCRs. Apart from these differences the activation mechanism of A_2A_R is similar to that of rhodopsin because Trp 6.48 is also moved and TM6 rotated. There are also similar rearrangements in β_2_AR and rhodopsin in TM5 and TM6, by movements of Phe6.44 towards Pro5.50 and Leu5.51 together with the movement of Ile3.40 away from Pro5.50 – such translocations are part of the transmission switch. Agonist binding in A_2A_R results in the relocation of Ser7.42 and His7.43 which, together with Thr3.36 in TM3, coordinate a part of the agonist. These interactions resemble the 3-7 lock between the protonated Schiff base of the retinal-Lys7.43 and Glu3.28, which is critical for rhodopsin activation. Although the TM3-agonist-TM7 interactions in the adenosine receptor are formed, rather than broken, upon activation, they could fulfill a similar role in arranging TM3 and TM7 in the active and inactive conformations [24].

The switches together with the hydrogen bond network between conserved residues, motifs and structural water molecules constitute an extended interface between different areas in GPCRs which facilitate the large movements linking ligand binding to cell signaling. Based on the recent crystal structures of inactive and activated, as well as constitutively active rhodopsin, one can elucidate the activation scheme of this protein and the role of particular switches as it was done by Standfuss *et al.* [122]. The structure of retinal in all-*trans* conformation, but unbound from Lys7.43, represents the active structure of rhodopsin nearly identical to the Meta-II state. This structure was published nearly simultaneously with the Meta-II rhodopsin structure with covalently bound retinal with and without GαCT (C-terminus of Gα subunit) [123]. The structures agree with each other in location of the main conserved amino acids. A covalently bound all-*trans* retinal behaves as a full agonist, whereas when unbound, it behaves as a partial agonist but maintains the critical interactions between the β-ionone ring and helices TM5 and TM6. The structure of a constitutively active mutant Glu3.28Gln [122] represents probably a trapped intermediate when retinal is either entering or exiting the binding site. The transition from an inactive to active state of GPCR includes large rigid motion of TM6. In the case of rhodopsin this is not a vertical hinge movement (named a global toggle switch) but a horizontal (in plane of the membrane) rotation of TM6 that leaves the shape of the helix intact. The characteristic bend of TM6 is imposed by Pro6.50 which is a part of the CwxP motif. The other highly conserved amino acid, Trp6.48, is tightly packed against retinal in the ground state of rhodopsin as it has a central role in the transmission switch model (previously called a rotamer toggle switch) of activation of these receptor. The structure of the Glu3.28Gln mutant places this residue 3.6 Å from its ground state position. However, no rotamer change is observed as it was proposed based on biochemical experiments and also computer simulations. Instead, Trp6.48 follows the retinal (its β-ionone ring) maintaining contact with the C_18_ methyl group. 

### 5.4 Tyrosine Toggle Switch (nPxxy Motif)

A region called the hydrophobic barrier (Fig. (**3A**)) separates the water mediated hydrogen bond network from the (d/e)Ry motif which is critical for G protein activation (Fig. (**2D-F**)). In the active Glu3.28Gln-GαCT structure, a rotation of TM6 disrupts the water mediated link between Trp6.48 and Ser7.45 and reorganizes the ground-state hydrogen bond network. The hydrophobic barrier opens and Tyr7.53 of the nPxxy motif, together with Tyr5.58, rearrange to fill the hydrophobic gap and to extend the hydrogen bond network towards the (d/e)Ry motif and GαCT peptide (Fig. (**3B**)). The role of this barrier in molecular switching was explained by Standfuss *et al.* [122] based on studies involving the crystal structure of rhodopsin with all-*trans* retinal in the binding site. The hydrophobic barrier was described earlier also by Schertler’s group [124] upon crystallization of inactive rhodopsin in a trigonal crystal form. A more extended motif nPxxy(x)_5,6_F was proposed by Fritze *et al.* [125] to explain the presence of the interaction between Tyr7.53 in TM7 and Phe7.60 in helix H8 in the inactive structure of rhodopsin (Fig. (**2D**). Such an interaction can additionally stabilize the inactive state of the receptor. However, in crystal structures of other GPCRs, such as the adrenergic and adenosine receptors (Fig. (**2E-F**)) such interaction is not seen despite the fact that Phe7.60 is present. It probably indicates that these receptors could be partially activated. 

The hydrophobic barrier consists of six residues between helices TM2, TM3 and TM6 (Leu2.43, Leu2.46, Leu3.43, Leu3.46, Met6.36 and Met6.40) and many of them are conserved in the rhodopsin-class of GPCRs. The rearrangement of hydrogen bonds is relatively minor but they directly link changes in the CwxP motif in the retinal binding pocket with the most conserved residues in TM1 (Asn1.50) and TM2 (Asp2.50) and nPxxy in TM7. On the cytoplasmic side, the rotation of TM6 opens the hydrophobic barrier allowing Tyr5.58 and Tyr5.53 to swing into the protein interior. They provide additional interactions with water molecules extending the hydrogen bond network toward the hydrophobic barrier to the (d/e)Ry motif at the cytoplasmic surface of TM3. The ionic lock involving residues in this motif, Glu3.49-Arg3.50 and Glu6.30, is broken and allows binding of GαCT peptide in a position that is occupied by TM6 in a ground state. Thus, rotation of TM6 and displacement of Trp6.48 results in a hydrogen bond network connecting residues from the retinal binding site to those at a cytoplasmic surface critical for activation of G protein.

Also the recently obtained A_2A_R-T4-lysosyme structure exhibits features of agonist induced rearrangements of a cluster of hydrophobic residues in TM3-5-6 near the binding site (Ile3.40, Leu5.51, Phe6.44 and Trp6.48) similarly to the active structures of β_2_AR [15, 108] and rhodopsin [122, 123]). However, although Tyr7.53 from the nPxxy motif is relocated towards the receptor center the relocation of TM6 is only 3Å which is much smaller than in active rhodopsin (6 Å), nanobody β_2_AR (8 Å) and in the complex with trimeric G protein (14 Å). These changes may be blocked by the presence of the fused lysozyme structure. However, the changes of residues close to the binding site suggest that this conformation resembles the Meta-I structure of rhodopsin which does not allow binding to G protein. Possibly in some GPCRs the full adaptation to G protein binding may be achieved in the presence of a G protein or other interaction partners that stabilize the cytoplasmic domain. 

### 5.5 The Elusive “Global Toggle Switch” 

The number of conserved motifs found in transmembrane helices of the Rhodopsin family receptors is significantly higher than in the other GPCR families (see Table **1** and Fig. (**1**)) indicating their potential role in receptor stabilization and activation. It was proposed that receptors of the Rhodopsin family most probably share the common mechanism of activation - the so-called "global toggle switch" [66]. According to this model the TM6 helix performs a vertical see-saw move around the central Pro6.50 residue during receptor activation induced by binding of an agonist. The upper part of TM6 is closing around the ligand, while the lower (near the intracellular surface) is opening to prepare for the G-protein binding. It was suggested that during activation by an agonist the rearrangement of TM3 and TM5 also takes place, though to a minor extent than in the case of the TM6 movement [24]. An accompanying kink in TM7 is induced by changes in the hydrogen-bond network between TM7 and TM1, TM2 and TM6 [21].

Using the metal ion site engineering techniques and based on the obtained distance constraints for β_2_-adrenergic receptor Elling *et al.* [126] developed the so-called “global toggle switch” mechanistic model. In this model Asp3.32 was an anchoring point for monoamine binding in TM3 helix. The authors engineered metal ion sites, which activated the receptor, between the extracellular parts of TMs. Copper and zinc ions alone and in complex with aromatic chelators acted as potent agonists in sites constructed between position 3.32 (Asp - known to bind ligand directly - or its mutation to His) and the Cys or His mutations of specific amino acids at TM6 and TM7 close to the binding site. To fulfill the distance constraints the residues involved in the orthosteric ligand binding pocket had to move closer to each other during receptor activation. In this model an inward movement of the extracellular segments, especially those of TM6 and, to some extent, TM7, was coupled to the well-established outward movement of the cytoplasmic segments of these helices. The authors suggested that the pivot points for these vertical seesaw movements are the highly conserved proline bends of the involved helices. Based on the present crystal structures of β_2_AR the global toggle switch must be modified because only a slight inward motion of the extracellular part of TM6 was detected. In rhodopsin there is even an increase of the retinal binding site and the same is in the case of A_2A_R – the binding site is smaller for antagonists regardless if they are smaller (caffeine) or bigger (ZM241385) than the agonist (adenosine). TM3, and not extracellular part of TM6 which is not moving, is responsible for this shrinkage of the binding site. There is an unusual bend on TM3 (close to Val3.32) in receptor structures with bound antagonists. This residue (located in the same position as extremely important Asp3.32 in receptors for monoamine ligands) may be now regarded as a part of the agonist/antagonist sensor. 

### 5.6 Role of Conserved Residues

Rhodopsin-like GPCRs lack a long N-termini except for PARs (protease-activated receptors which do not need agonist-binding to be activated) with an N-terminal thrombin-cleaved part releasing a tethered ligand, LGRs (GPCRs containing LRRs – leucine-rich repeats) interacting with glycoproteins and LDLa (a low-density lipoprotein receptor class A). In most Rhodopsin-like GPCRs an agonist interacts with extracellular loops and the TM region. Although sequence diversity in the TM region is quite high even within the Rhodopsin family the motifs involved in the activation mechanism are well conserved, i.e. (d/e)Ry, CwxP and nPxxy. In Table **1** we indicated all conserved residues in the Rhodopsin family of receptors and underlined these which are believed to be involved in molecular switches. The residues are also visualized on the topological scheme of GPCR (7TM receptor) in Fig. (**1**). 

In TM1 the most conserved residue is Asn1.50, involved in a structural water-mediated hydrogen-bonding network between TM1, TM2 (Asp2.50), TM6 (Trp6.48) and TM7 (Asn7.45, Ser7.46, Asn7.49, Tyr7.53). The Asn 1.50 residue is an arguable element of the receptor activation process, namely the TM3, TM5 and TM7 movements [21]. The conserved proline residue in TM2 [127], either in position 2.58 (e.g. in a recently solved CXCR4 structure) or 2.59 (rhodopsin and adenosine receptors), which induces a helix kink in the first case or a helix bulge in the latter, is crucial for the ligand binding, but does not play a significant role in the receptor activation. As in most of GPCRs cysteine residues are highly conserved in the Rhodopsin family and form disulphide bridges stabilizing the receptor structure. The most important cysteine pair is Cys3.25 connected with Cys in EC2. Glu3.28, which is present only in the Rhodopsin PDB structure, serves as a counterion with the protonated Schiff base in 11-*cis*-retinal and possibly stabilizes an inactive state of opsin [128]. Asp3.32 with Trp7.40 and Tyr7.43 (instead of Lys7.43, more frequent in the Rhodopsin family – see Table **1**) are a unique fingerprint only for biogenic and trace amine receptors (a G2 group) not shared by any other Rhodopsin-like GPCR. Asp3.32 with Tyr7.43 were proved to form the TM3 – TM7 ionic lock stabilizing the unbound, inactive state of the receptor [129]. Asp3.32 is believed to serve as a counterion for the amine groups of native ligands [130]. A residue in the 3.36 position is not well-conserved in Rhodopsin-like GPCRs, however, it was confirmed experimentally by site-directed mutagenesis, that this residue interacts with Trp6.48 and stabilizes the inactive state not only in the β_2_-adrenergic receptor (Val3.36, van der Waals interactions) [18] but also in serotonin receptors (Ser/Cys/Thr3.36, hydrogen-bonding) [129], the opsin subclass (Gly3.36) [131] and cannabinoid receptors (Phe3.36 aromatic stacking with Trp6.48 – a rotamer toggle switch) [132, 133]. Glu/Gln3.37 is a key residue in agonist binding to LH and TSH receptors [134]. A well-conserved Leu3.40 residue which is close to Pro5.50 before activation and becomes distant after, plays a key role in the TM3 – TM5 movement [24]. 

A well-conserved Trp4.50 is a cholesterol binding-site which is visible in the structure of human β_2_-adrenergic receptor [59]. Phe5.47 stacks against Phe6.52 and possibly interacts with agonists [88] but still little is known about its function. Phe6.44 together with Phe6.52, Leu3.40 and Leu5.51 is forming a hydrophobic and aromatic cluster around Trp6.48 involved in conformational rearrangements of TM5 [24, 129]. Pro6.50, like Pro7.50, produces a helix kink around which TM6 performs movements during activation. Tyr7.53 in the nPxxy motif interacts with Phe7.60 in helix H8 and forms a molecular switch between active and inactive conformation.

### 5.7 Role of Extracellular Loops in Ligand Binding and Switching

The extracellular loops also have an influence, although sometimes transiently, on ligand binding and could participate in some types of molecular switches. The recent crystal structures of GPCRs revealed that the part of the receptor extending from the orthosteric ligand-binding site in the transmembrane domain to the cytoplasmic side is highly structurally conserved. In contrast, the extracellular surface of GPCRs is substantially diverse and, therefore, could be a target of highly selective drugs. However, still little is known about the coupling of the extracellular surface to the ligand-binding compartment. Bokoch *et al.* [135] used NMR spectroscopy to investigate ligand-specific conformational changes around a salt bridge linking extracellular loops EC2 and EC3 (Asp192-Lys305) in β_2_AR. It was demonstrated that small-molecule hydrophilic drugs that bind within the transmembrane core and exhibit different efficacies towards G-protein activation (agonist – formoterol, neutral antagonist – alprenolol or unliganded receptor, and inverse agonist - carazolol) also stabilize distinct conformations of the extracellular surface. Such conformational coupling supports the possibility of an efficient allosteric action of specific drugs targeting this diverse surface with high subtype selectivity. Although the specific salt bridge used to monitor these conformations may not be present in other GPCRs it is likely that ligand-induced changes at the extracellular surface are relevant for other family A GPCRs. 

In adrenergic receptors only one residue in the EC2 loop can interact with ligands in the binding site: this is Phe201 in β_1_AR and an equivalent residue, Phe193, in β_2_AR. A disulphide bridge located two residues away from that phenylalanine residue keeps the proper conformation of EC2 and assures that such interactions with the ligand will be preserved. In the recent crystal structures of these receptors with agonists the ligands do not appear to interact with this residue, however, if we examine the possible entrance way of the ligand into the receptor binding site we can notice that the ligand may interact transiently with Phe193/201. Therefore, it is possible that Phe 193/201, together with other residues from the extracellular loops, could participate in the action of molecular switches. The binding sites of other receptors with diffusible ligands are more spacious so the binding of a ligand is more straightforward and could be done without a transient binding to the EC2 loop. However, even in those receptors the ligands can interact with the EC2 loop. An interesting case is a recent crystal structure of a chemokine receptor CXCR4 with a peptide ligand CVX15 consisting of 16 amino acids [33]. Binding of that ligand involves a large number of residues from the EC2 loop and also from the N-terminus. However, because of the lack of structures of CXCR4 with agonists, there is no direct data on the involvement of extracellular surface residues in molecular switching. Even in the case of rhodopsin the EC2 loop, which tightly covers the retinal binding site, is moving upon retinal isomerization and this movement, from the retinal-binding site, is coupled to the rotation of TM5 and to the inward motion of the TM6-EC3-TM7 segment [136].

The hydrophobic ligands, like retinal in the case of rhodopsin, are probably entering the receptor binding site directly from the membrane. There are two openings of the retinal-binding site in the crystal structure of opsin (ligand-free rhodopsin) [89] one between the extracellular ends of TM5 and TM6, and another between TM1 and TM7. It was suggested that the opening between TM5 and TM6 could be selective for the uptake of 11-*cis*-retinal. The smaller opening between TM1 and TM7 could be a site for the release of all-*trans*-retinal. A putative external binding site for retinal is possibly located in the kink region between TM7 and H8 closely to palmitoyl chains [137]. The mechanism of retinal movement is potentially significant for vision in the regeneration pathway, the disorders of which have been associated with different forms of blindness. In the recent structure of CXCR4 there is also a gap between EC ends of helices TM5 and TM6 which is filled up by lipids. The hydrophobic ligands of this receptor could potentially enter the receptor binding site through this hole. However, the open question remains which residues could be responsible for sensing the ligand type and which ones participate in switching mechanisms. 

## 6 ACTIVATION SCHEMES

The recent period proved to be very fruitful in GPCR research – many new structures were crystallized and, what is even more important, first time with agonists (β_1_- and β_2_-adrenergic [104, 108] and adenosine [101] receptors and recently also rhodopsin with all-*trans* retinal [122, 123]). This greatly facilitated elucidation of the activation scheme of these receptors. Now, another breakthrough has been made i.e., the long awaited crystal structure of the complex of GPCR with the whole G protein is available [96] and one can expect that similar structures of other GPCRs will be also available. Possibly, a new and exciting mystery to be solved is the allosteric influence of dimers on the process of activation. In a very interesting review compiled by Deupi and Kobilka [42] about the energy landscapes of GPCR activation it is shown how structural changes of GPCRs during their activation can be visualized on energy landscapes. Because of high structural similarity of all crystallized GPCRs, the activation scheme is probably similar for all GPCRs so it is suggested that the receptors are passing through the same stages during the activations process. This similarity is much higher in the cytoplasmic side of the transmembrane bundle. This region contains residues involved in receptor activation and binding of a G protein. Similar conformational changes underlying activation of GPCRs are also deduced from numerous biochemical and biophysical experiments. Probably also a sequence of events is nearly identical and involves the following steps: first, small changes in TM5 and TM7, then a large change in TM6, and then neutralization of Asp3.49 in the (d/e)Ry motif (Fig. (**2G-I**)). Two-dimensional energy landscapes seem to be more advantageous over one-dimensional energy plots but currently too little is known for precise construction of such plots. Possibly new crystal structures supplemented by long molecular dynamics simulations will help in designing so useful but also elegant and eye-catching charts. 2D or even 3D energy plots make possible dissection of the reaction pathway into discrete non-sequential conformational changes providing alternate routes of activation through the energy landscape. In this way some events may be skipped for some ligands and a full or partial activation state can still be achieved.

### 6.1 Two Types of Activation Paths

The substantial amount of data obtained from rhodopsin and also adrenergic receptor activation can serve as a framework to reveal activation of other GPCRs. There are some variations, though. It is suggested [42] that the β_2_AR is not trapped in a fully inactive conformation in the absence of agonist but its internal flexibility allows the receptor to explore different conformations. This may suggest a shallow energy landscape with several conformational states separated by relatively low energy barriers. On the contrary, for rhodopsin (and similarly activated GPCRs) it is supposed that binding of agonist is invoking an induced fit of the receptor structure. Therefore, agonists have to bind to the receptor with high affinity and this high binding energy is used to initiate conformational changes (“jump”) over the highest initial barrier of energy. Retinal isomerization in rhodopsin provides such high energy. In case of other receptors (although being classified in the Rhodopsin-like family because of their sequence) the ligands have relatively low affinity and rapid dissociation rates; these features may indicate a conformational selection procedure of activation. After ligand binding the sequence of events during receptor activation is regarded as being similar in all these receptors. Any differences are rather not in a number of steps required for full activation but rather in the size of energy minima depths. The well-known example of differences in the activation scheme is the existence of an open ionic lock in crystal structures of β_1_AR and β_2_AR adrenergic receptors with antagonists and inverse agonists bound that may suggest a very low energy barrier for opening of this switch. The late stages of β_2_AR activation, which are supposed to be analogous to achieving the Meta-II stage in rhodopsin activation, involve a similar set of conformational changes, i.e. rearrangement of TM6 and neutralization of Asp3.49 in the (d/e)Ry motif of TM3. According to the above two schemes of activation, the partial agonism can be also explained in two ways. For those GPCRs from which partial agonists dissociate faster than full agonists, not all binding events last long enough to promote activation of the G protein. Another possibility is that the partial agonists stabilize different intermediate conformations that lead to alternate activation pathways and to non-optimal G protein activation. Particular steps can be achieved either by induced fit upon binding of a ligand or by conformational selection but the achieved equilibrium states would be completely indistinguishable. It is suggested that the induced fit mechanism is present in rhodopsin and angiotensin AT_1_ receptor whereas β_2_AR may function by selecting specific receptor substates by the ligand.

## 7 THEORETICAL STUDIES ON THE ACTION OF MOLECULAR SWITCHES

### 7.1 Single TM Studies

One of the first computational studies aimed at GPCRs switches was done in 2001 by Ballesteros *et al.* [113] who simulated the disruption of the TM3-TM6 ionic lock. Simulations presented in that work concerned only TM6 and short MD runs to simulate the bending of TM6 at the Pro6.50 position were performed. From those computational studies combined with experimental mutations of the Glu6.30 residue it was concluded that a conformational rearrangement of TM6 is highly correlated with the extent of constitutive activity of different mutants. A similar approach was used later to study the conformational switch in the 5-HT_2C_ receptor [138]. Again, a combined computational-experimental study showed that a conserved Tyr7.53 residue is interacting with the conserved Tyr7.60 (in helix 8) contributing to the switching of the receptor among multiple active and inactive conformations. Although the ‘ionic lock’ is still regarded as an important switch it can be open in crystal structures of GPCRs even with antagonists and inverse agonists. Currently, only in the inactive and partially active rhodopsin structure (batho and lumi intermediates) that switch is closed [139]. 

A similar approach was presented in the 2002 paper by Shi *et al.* [140]. In this work Monte Carlo techniques were used to sample rotamer changes among the X6.47- Trp6.48-Phe6.52 residues of the human β_2_AR model of TM6. The results show a high correlation between the conformation of side chains of these residues and the helix kink at the Pro6.50 position, which was consistent with the experimental data for rhodopsin [141]. While it was clear that simulations on isolated helices could not predict the global interaction and changes in GPCRs, the results showed the usefulness of computational methods for studying ionic locks.

The same ionic lock has been studied in the 5-HT_2A_ system in the 2002 paper by Visiers *et al.* [142]. Here, authors decided to concentrate on the electrostatic properties of the conserved residues. By solving the Poisson-Boltzman equation to obtain electrostatic potentials of the different conformers of important residues on the TM3-TM6 model, it was found that Glu3.49 may undergo protonation upon activation of the GPCR. The activation of the protein has been also explained as a change in the kink at Pro6.50 which allows the ends of TM3 and TM6 to move away from each other. Based on the computational results it was suggested that selected, single-point mutations (Glu6.30Asn, Glu6.30Gln, Glu6.30Leu) would disrupt the electrostatic interactions of the (d/e)Ry motif with this residue. This prediction was confirmed later by the results of site directed mutagenesis, where it was shown that a neutral residue at the 6.30 position increases the activity of 5-HT_2A_ in the absence of the ligand, similarly to the human β_2_AR case.

### 7.2 Studies on a Complete GPCR Model

#### 7.2.1 Investigations of the Ionic Lock Switch

The most prominent method to study the dynamics of GPCRs is nowadays Molecular Dynamics (MD) of the complete GPCR model. One of the first MD simulations focusing on TM3-TM6 ionic locks was performed in 2002 by Greasley *et al.* [143], who simulated the β_1_AR model based on the rhodopsin structure of Palczewski. The methodology used the united atom model and included a large number of short (150 ps) MD runs of the protein only (without environment), using NOE constraints to preserve the α-helix structure of TMs. The short time of simulations was due to the limited computational resources available at that time. The results showed a very high stability of the Arg3.50-Glu6.30 salt bridge. Combined with experimental mutational data (Glu6.30 mutations that weakened this ionic lock constitutively activated the receptor) the results showed that the transition from the inactive to active state of α_1b_AR involves a rearrangement of helices TM3 and TM6. The structure of α_1b_AR is still not available but predicted movements were confirmed by crystal structures of activated β_1_- and β_2_-adrenergic receptors.

**Fig. (2):**
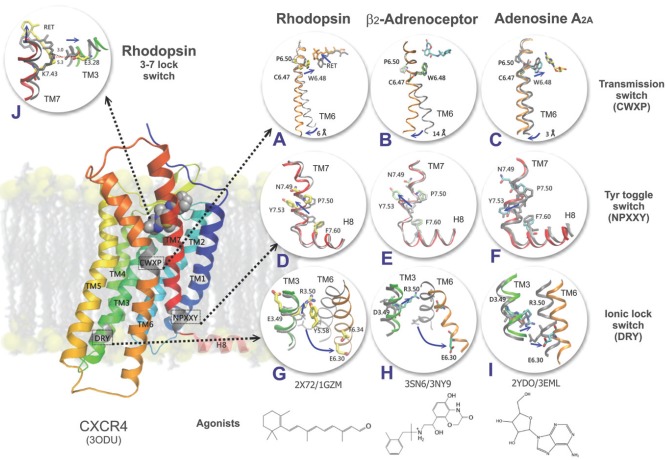
The action of molecular switches in GPCRs. Four switches are shown: transmission switch, tyrosine toggle switch, ionic lock, and 3-7 lock. They are shown based on the crystal structures of rhodopsin, β_2_AR and A_2A_R with agonists and antagonists/inverse agonists. Their id numbers from Protein Data Bank are provided – first number for inactive and second for active receptor. Additionally, the structural formulas of agonists from the crystal structures of active receptors are shown. The general scheme of GPCR structure is shown based on the crystal structure of chemokine receptor CXCR4 with a small ligand. Blue arrows in circular panels indicate motions of receptor structure during action of particular switch. Inactive receptor structure is shown in gray while active one in color. The residues are numbered according to the Ballesteros-Weinstein numbering scheme [90].

In the same year Rohrig *et al.* presented their work in which a full-atom model of the bovine rhodopsin has been immersed in a layer of a n-octane mimicking lipid bilayer [144]. The goal of these simulations was to show the effect of retinal *cis*-to-*trans* isomerization on the dynamics of the protein. Indeed, it was found that retinal isomerization serves as a trigger for propagation of the signal to the surrounding helices. Despite the limited simulation time authors were able to see some rearrangements in TM6 and to a lesser extent also in TM4 and TM5. They have also noticed cleavage of selected hydrogen bonds in these helices, however the ionic lock remained stable over the course of simulations. In 2003 a team led by Thomas B. Woolf applied MD simulations to the full atom bovine rhodopsin model including DOPC lipid membrane and surrounding by water molecules [145]. Their 40 ns simulation (a huge amount in those days) became a basis for a number of similar MD simulations on GPCRs in the following years. Similarly to the Rohrig studies this work concentrated on the rhodopsin vicinity and the changes in residues interacting directly with the rhodopsin ligand. The authors noted a strong interaction between residues forming the ionic lock (Arg3.50-Glu6.30) throughout the whole simulation time (as in Fig. **2G**).

A similar approach as in the Crozier work has been applied to a large number of GPCR MD studies, although the advances in computational powers allowed to lengthen the simulation scale to tens and hundreds of nanoseconds. One of the interesting works showing the stability of various locks was the MD investigation of the opioid receptors models. In the μ-OPR opioid receptor there is no glutamic acid residue in the 6.30 position, but the TM3-TM6 lock is still present in the modeling studies due to the hydrogen bond between Arg3.50 and Thr6.34 [117, 118]. In the most recent simulations this lock remains unbroken even in the presence of selected antagonists or agonists [119, 120], most possibly because of a rather short length of simulations (nanosecond time scale) compared to the time needed for activation of the receptor (milliseconds). Binding of antagonists has also no effect on the Trp6.48 rotamer switch which remains in the initial, rhodopsin-like vertical position. Interestingly, μ-OPR agonists toggle the Trp6.48 position to a horizontal one which, during simulations, forces the change in positions of aromatic residues around the highly conserved Pro6.50. It is also worth noticing that the mutation of Leu6.30Glu in the μ-OPR system inactivates this receptor [146]. Simple calculations of the electrostatic interactions for two helices (TM3 and TM6) have shown that the Leu6.30Glu mutation enhances the hydrogen bond network around the mutated residue and stabilizes the inactive state.

#### 7.2.2 Breaking of the 3-7 Lock Switch

During the MD simulations of μ-OPR it was noticed that another switch, the 3-7 lock, a link between TM3 and TM7 (a hydrogen bond Asp3.32-Tyr7.43), remains stable for the apoprotein simulations and in the presence of antagonists. The presence of agonists, however, forced the hydrogen bond to break [118]. For one of the agonists it was also shown that rotation of Trp6.48 was linked to the cleavage of the Asp3.32-Tyr7.43 hydrogen bond and occurred within 1 ns after its breaking. Later, MD studies have shown that a similar cascade of events is likely to occur also upon agonist binding and activation of δ-OPR and κ-OPR [119]. Recently, a structurally similar κ-OPR agonist and antagonist pair, guanidinonaltrindole (GNTI) compounds, were investigated by molecular dynamics simulations [120]. 5′-GNTI is an antagonist while 6′-GNTI is an agonist. Ligands were relaxed in the receptor binding site by the simulated annealing routine. During a series of MD simulations of the ligand-receptor complexes in DPPC membrane, the 3-7 lock was broken when the agonist was bound, but remained unbroken upon binding of the antagonist. The hypothesis of 3-7 lock breaking on the agonist binding still awaits confirmation, since no experimental structure of any opioid receptor is available at this time.

#### 7.2.3 Beyond Classical MD Techniques

In 2009 Provasi *et al.* studied the dynamics of the δ-OPR system using a metadynamics approach [147]. This approach allows for an efficient exploration of multidimensional free energy surfaces of GPCRs (and other biological systems) by adding a history-dependent bias to the interaction potential of the system. The required microsecond-scale well-tempered metadynamics has been achieved using the united-atom model for lipids (DPPC and cholesterol molecules) and the full-atom model for protein and ligand. It allowed the authors to suggest a preferential entry pathway of the NLX antagonist, starting from the δ-OPR surface and ending in the proper binding pocket of the GPCR, and to evaluate the bonding constants for the ligand. The observed binding pocket was extremely close to the previously predicted one and the starting structure for their system had all the known locks including the Arg3.50-Thr6.34 of TM3-TM6 lock (or ‘3-6 lock’ instead of ‘ionic lock’ since no salt bridge can be formed in this case) and the Asp3.32-Tyr7.43 of 3-7 lock. 

It is also worth mentioning that other computational techniques have been used to model the activation of rhodopsin and they gave similar results. In 2006 Niv *et al.* used an Elastic Network Model to study the inactive form of rhodopsin [148]. In this model a harmonic potential with a single force constant accounts for pairwise interactions between all Cα atoms within a certain cutoff distance. The analysis of the structural relation of the inactive normal modes to the transition vectors towards the active conformations has been discussed. It has been found that the active form of rhodopsin should be characterized by structural changes in TM5-6-7, while helices TM1-2-3-4 were shown to be the most stable ones which were confirmed later by crystal structures of Meta-II rhodopsin. Niv *et al.* also predicted the rotamer toggle switching of Trp6.48 and they claimed that it was in agreement with the early spectroscopic data [149]. However, this was not confirmed by later crystal structures of opsin and also rhodopsin with all-*trans*-retinal bound [123] and unbound but still present in the binding site [122]. The spectroscopic properties of Trp6.48 really change during activation of rhodopsin but it is a result of large movement of cytoplasmic part of TM6 and smaller movements of adjacent helices so a local environment of Trp6.48 is altered even if a rotamer of Trp6.48 itself does not change. 

Much more detailed approach to the study of ionic-lock induced activation of GPCRs has been proposed in the work of Balamaran *et al.* [150]. In this work the all-atom force field has been used to evaluate the relative stability of various point-mutations in β_1_-adrenergic receptor. The results showed good correlation with the experimental data for over 90 single and multiple point mutants of this protein. It was found that Tyr5.58Ala and Val5.61Ala mutations stabilize the Arg3.50–Glu6.30 ionic lock, while Phe7.48Met mutation alters the interaction between the conserved NPxxY motif of TM7 and TM8. 

#### 7.2.4 Consequences of the Ionic Lock Instability and TM Movements

The first experimental structure of GPCR, opsin, provided structural data which confirmed the importance of molecular switches and ionic locks [89]. While some of the structures of the early photoproducts of rhodopsin showed only an increased Arg3.50-Glu6.30 distance [139], in the opsin structure these residues are no longer interacting with each other. Arg3.50 is released from the Glu6.30 and Glu3.49 interactions and engages with Tyr5.58 on TM5, an interaction that was not suggested before. At the same time a new interaction between Lys231 and Glu247 is formed to stabilize TM5-TM6 interactions. Also, the Tyr7.53 of the nPxxy motif (TM7) aromatic stacking interaction with Phe7.60 (H8) is broken due to a different orientation of the helices. On the other hand the Trp6.48 toggle switch is in exactly the same position as in the inactive rhodopsin, even though it was indicated by NMR studies that it must change its position and interaction partners during activation [151]. 

A very similar structure of the active opsin, albeit with a different Trp6.48 rotamer, has been predicted computationally by Bhattachary *et al.* at the same time [152]. The authors started from the inactive (dark) state and have predicted TM conformational changes that are induced by the isomerization of 11-*cis* retinal to all-*trans* retinal with good accuracy. In another study Hornak *et al.* used nanosecond MD guided by NMR distance restraints to simulate the activation of rhodopsin [153]. Results of this work were also in agreement with experimental data and showed the coupling of retinal isomerization to the motions of helices and activation of the receptor which proceeds *via *a series of multiple switches.

To solve this inconsistency in behavior of Trp6.48, a MD simulation of β_2_AR and bovine rhodopsin systems combined with mutational analysis of the ghrelin receptor was performed [154]. The 8 ns simulation of inactive rhodopsin with 11-*cis*-retinal present, showed no change in the Trp6.48 conformation. The removal of retinal, however, allowed this residue to change its rotameric state. After additional 9 ns of simulation Trp6.48 established a moderately strong and not very stable interaction with Phe5.47 on TM5. In the β_2_AR case removal of its ligand (carazolol) did not result in large conformational changes of Trp6.48 and no additional interactions have been observed. Additional data obtained from metal ion site engineering confirm the close proximity of these two residue. Mutational data from ghrelin receptors experiments show, on the other hand, that both of these residues are important for constitutive activity and agonist-induced efficacy. Authors suggest that the ionic lock may be one of the several molecular switches that form an allosteric interface between the TMs performing global toggle switch movements that mediate the intramolecular signal which leads to G protein activation. However, since ~30% of GPCRs lack the Trp6.48 residue, it may not be a part of the general activation pathways for 7TM receptors.

The problem of the lack of interaction between Arg3.50 and Glu6.30 in the β_2_AR crystal structure, despite their presence in the sequence, has been addressed in a 2009 paper by Dror *et al.* [155]. For this system there was also a variety of biochemical evidence suggesting that an ionic lock between those residues is formed in the inactive state [143]. It was suggested that the broken lock may be a consequence of the techniques used to stabilize β_2_AR for crystallization or of the binding of carazolol or timolol, which might reflect the ability of some partial agonists to induce signaling through disruption of this interaction. An all-atom simulation of β_2_AR over 10 microseconds showed that an ionic lock forms reproducibly both in apo β_2_AR and in the carazolol-bound system. Interestingly, the ionic lock had a tendency to break in simulation every few hundred nanoseconds and the conformation with this lock broken remained stable for tens of nanoseconds. In conclusion the authors suggested that the inactive β_2_AR alternates between several major conformations with the ionic lock present and a few minor conformations without the ionic lock. Their long, microsecond simulation time allowed to describe the inactive state of this GPCR as an ensemble of states.

Almost at the same time another MD work on β_2_AR and β_1_AR appeared [156]. 500 ns MD runs for carazolol-bound β_2_AR model and cyanopindol-bound β_1_AR model showed the formation of the ionic lock, which remained stable during the stimulation runs, in both systems. In this work no breaking of the ionic lock has been observed; however, the timescale of the simulations might have been too short for such event to occur. Interestingly, water-mediated interactions Trp6.48-Asp2.50 and Asp2.50-Asn7.49-Tyr7.53 have been observed, some of which have been suggested to be important in activation of the thyrotropin receptor. The water-mediated hydrogen bonds also remained stable during the simulation runs. The results of this study were different than the earlier theoretical investigation of the impact of ligand binding on the conformational state of the protein. Bhattachary *et al.* investigated the perturbations in the helical rotational orientations induced by ligand binding in the TM region of the β_2_-adrenergic receptor [157]. They found that norepinephrine (full agonist) and dopamine (a weak partial agonist) break the Arg3.50–Glu6.30 ionic lock and engage the Trp6.48 rotamer toggle switch, while salbutamol (a partial agonist) only breaks the ionic lock and catechol (a very weak agonist) only switches the rotamer toggle. 

A longer, 1.02 microsecond simulation of β_2_AR presented by Romo *et al.* allowed the researchers to show the interconversion between ionic lock substates [158]. Similarly to previous results authors showed that the Arg3.50-Glu6.30 ionic lock is able to break and reform in the wild-type β_2_AR simulations. The lock-breaking event is followed by the reorganization of the cytoplasmic end of TM6 through a clockwise rotation of the helix and movement of the end of TM5 away from TM6. These events were in agreement with the predicted activation models of this system. In 2011 Moukhametzianov *et al.* followed the work of Dror and showed that also β_1_AR may have at least two distinct conformations in the inactive state [159]. Their new X-ray structures of this receptor show that TM6 and IC3 loop (connecting TM5 and TM6) may have a bent form, stabilized by previously unseen interactions Met281-Leu282 and His286-Gln237. They also noticed that the new TM6 conformation positions Arg3.50 and Glu6.30 only 3.8 Å apart, which is a distance significantly longer than in the rhodopsin case (with the ionic lock), but also much shorter than in the other conformation of TM6 (where the distance is 6.2 Å).

Experimental structures of active states of rhodopsin and β_2_AR have shown that MD predictions of the behavior of ionic locks during activation were generally accurate. The constant development of computational methods and the more and more powerful computers allow us now to reach microsecond simulations [107, 160-162] and obtain information about the general energy landscapes of GPCRs. Such theoretical energy landscapes and activation pathways, even though simplified to include only one or a few coordinates, give a very good means to look inside the GPCR activation mechanisms. The latest experimental results, e.g. highlighting the role of the highly-conserved Tyr5.58 in the stabilization of the active state of rhodopsin [163] are perfect examples of data and events that may be simulated by computational methods to gain more understanding of their mechanism of activation/inactivation and molecular processes involved in them.

## 8 DRUG DESIGN

Development of selective drugs for GPCRs is challenging because (1) there is a high degree of homology among many closely related receptor subtypes that can regulate diverse physiological functions; (2) a single receptor may couple to more than one G protein or even signal through G protein independent pathways; (3) a receptor can be regulated by multiple allosteric ligands (small molecules but also proteins including GPCRs – what implicate allosteric influence by dimerization or oligomerization either homomers or heteromers; (4) predominant signaling behavior of GPCRs may differ in different cells or organs. 

Despite the progress which led to insights into the three-dimensional structures of GPCRs in both active and inactive states, the process of developing a drug for a particular GPCR target has become more complex, time-consuming and expensive [164]. Detailed characterization of agonist and antagonist binding behavior provided insight into the allosteric effect of G proteins on receptor structure and agonist binding affinity. The efficacy of ligands for activating the arrestin pathways can differ from those that activate G proteins. Some ligands possess a complex pharmacological behavior acting as agonists and simultaneously antagonists or inverse agonists depending on the pathways they activate and inactivate. Carvedilol is an inverse agonist for β_2_AR activation of Gs but a partial agonist for β_2_AR activation of arrestin [165]. The complexity of GPCR signaling pathways and ligand efficacy profiles complicate the process of drug discovery. Moreover, specific receptors might exhibit cell type-specific signaling as a consequence of the cell-specific complement of different proteins: signaling, regulatory and scaffolding.

As can be seen from crystal structures the structural diversity of GPCRs is much greater for amino acids lining the pathway into the ligand binding pocket. Although these amino acids do not make direct contact with a bound ligand the size of carazolol, they could contribute to initial transient interactions between the ligand and receptor that affect the ligand association rate. These residues might also play a role in binding larger ligands that extend into the vestibule of the binding pocket, such as the long-acting β_2_AR agonist, salmeterol, that is used in the treatment of asthma [164]. The recent crystal structure of the chemokine CXCR4 receptor with an antagonist in the form of a peptide ligand, which extends from the receptor binding side toward the extracellular surface, show such kind of binding for the first time for GPCRs (PDB id 3OE0) [33]. Although many aspects of GPCR function can be explained by a simple two-state model, evidence from biophysical and functional studies support a multistate model in which ligands stabilize a specific conformational state or set of states [42] making the complexity of GPCR signaling similar to the microprocessor work [166]. 

## 9 CONCLUSIONS

Investigations of molecular switches in the superfamily of GPCRs is extremely challenging but may be truly rewarding because the detailed mechanism (or mechanisms) of activation of these receptors could help to design highly selective drugs acting not even on a single receptor subtype but on a single pharmacological receptor subprofile. Because of the intrinsic instability of GPCRs resulting in their multiple functionality, the investigations must proceed *via *elucidation of multiple structures with inverse agonists, antagonists and agonists, possibly also with trimeric G proteins and arrestins. Dimerization is a separate large issue currently unresolved but possibly the allosteric action of GPCRs *via *dimerization is the most common mechanism influencing receptor functioning. GPCRs are the biggest and one of the most mysterious single group of molecular targets for drugs, therefore, one can be sure that studies on their structures and mechanisms will be continued with an increasing pace. 

## Figures and Tables

**Fig. (1):**
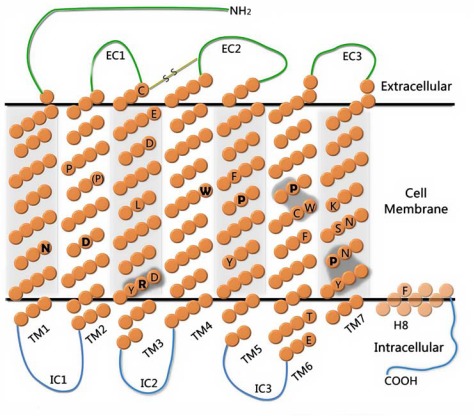
General scheme of topology and location of conserved residues in Rhodopsin-like GPCRs. Number of residues and their locations in each TM is based on chemokine receptor CXCR4 (H8 is not present in the crystal structure so it is shown transparent). Residues in bold are the most conserved in each TM. Sequence motifs are shown as gray areas. An alternative position of proline residue in TM2 is denoted by (P). Detailed description of figure is done in main text.

**Fig. (3):**
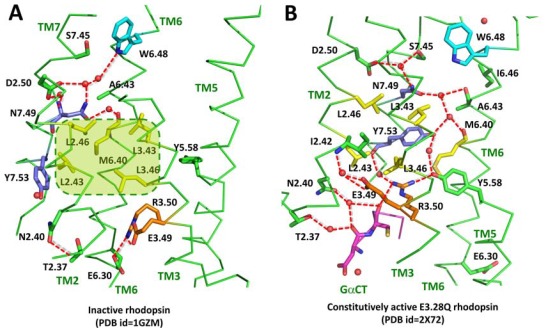
Rearrangement of hydrogen bond network in rhodopsin during its activation. A. The crystal structure of inactive rhodopsin (Protein Data Bank id 1GZM). B. The crystal structure of constitutively active Glu3.28Gln mutant of rhodopsin with all-*trans* retinal unbound from Lys7.43 but still present in the binding site (Protein Data Bank id 2X72). Both structures include water molecules (shown as red spheres) which participate in hydrogen bond network. In the inactive rhodopsin there is a hydrophobic area consisted of five residues located in helices TM2, TM3 and TM6 (in yellow) which form a hydrophobic barrier (area in green) separating residues in CwxP (cyan) and nPxxy (blue) motifs from those of (d/e)Ry motif (orange). In the activated rhodopsin a rotation of TM6 disrupts the water mediated link between TM6 and TM7 and reorganizes the hydrogen bond network. Two tyrosine residues Tyr5.58 and Tyr7.53 reposition and fill the uncovered gap between TM3 and TM6 to extend hydrogen bond network toward (d/e)Ry motif and a fragment of G protein G􀀁CT (pink). Figure is based on [122]. The residues are numbered according to the Ballesteros-Weinstein numbering scheme [90].

**Table 1.** Characterization of GPCRs families. Important motifs are bolded and residues involved in molecular switches are underlined. Uppercase letters in the motifs indicate completely conserved positions, lowercase indicate well-conserved positions (>50%) and x indicates any amino acid. The residues are numbered according to the Ballesteros- Weinstein numbering scheme [90]

Family	Orthosteric binding site	Overall sequence diversity	Well-conserved motifs/residues and molecular switches based on [21, 69, 127]											
			No. of conserved res. in TM regions	N-terminus	TM1	TM2	EC1	TM3	TM4	EC2	TM5	TM6	TM7	Cterminus & Helix 8
Rhodopsin	TM region, EC loops, less frequently: N-terminal region (LRR, LDL , PAR)	high	25	Cys	Asn1.50	Asp2.50 Pro2.59 (Pro2.58) a	-	Cys3.25 Glu3.28 b Asp3.32 Glu/Gln3.37 c Leu3.40 Asp3.49 (Glu3.49) Arg3.50 Tyr3.51 Tyr3.60 **(d/e)Ry**	Trp4.50	Cys	Phe5.47 (Asn/Asp5.47 ) c Pro5.50 Tyr5.58	Glu6.30 Thr6.34 Phe6.44 Cys6.47 Trp6.48 Pro6.50 **CwxP**	Lys7.43 (Tyr7.43) Asn7.45 Ser7.46 Asn7.49 Pro7.50 Tyr7.53 **nPxxy**	Phe7.60
Glutamate	N-terminal region (VFTM, SUSHI)	low	94	Cys	Aliphatic, aromatic, polar	Aliphatic, aromatic, polar	-	Cys Aliphatic, aromatic, polar, charged(+)	Cys Aliphatic, polar	-	Cys Aliphatic, polar, aromatic	Cys Aliphatic, polar, aromatic **wl**	Cys Aliphatic, aromatic, polar, charged(+) **pkxy**	-
Adhesion	N-terminal region (i.a. GPS, HBD)	high	6	Cys **cxCxhlt**/s	-	Polar, charged(+)	Cys	Cys	Aliphatic	Cys	-	Cys Aliphatic, polar	Cys	-
Secretin	N-terminal region, EC loops, TM6	high	33	Cys**CnxxwDxxx xCW rxCxxxGxw**	Aliphatic, polar	Aromatic, aliphatic, charged(+)	Cys	Aliphatic, aromatic, polar, charged(-)	Aromatic, aliphatic, charged(+)	Cys	Aliphatic, polar	Aliphatic	Aromatic, aliphatic, polar	-
Frizzled/Taste2	N-terminal region (Wnt binding domain d ), EC loops	low/high e	81 in Taste2: 103	Cys	Aliphatic, polar, aromatic	Aliphatic, polar, aromatic, charged(-) f	Cys T/M, I/V f**NxWaVtnH**^f^	Aliphatic, polar, aromatic	Aliphatic, polar, aromatic, charged(+)	Cys G/D f S/N> f	Aromatic, aliphatic	Aliphatic, polar, aromatic	Aliphatic, polar, aromatic, charged(+)	L/R

a Only in the G1 group according to Chabbert’s classification [32].

b Only in the Rhodopsin PDB structure.

c Only in LGR receptors (Leucine-rich repeat-containing GPCRs). LGR receptors are members of the G3 group according to [32].

d Not in Taste2 receptors.

e Low – Frizzled, high – only in the N-terminal region of Taste2 receptors.

f Only in Taste2.

**Table 2.** Summary of All Available Crystal Structures of GPCRs (Based on [61])

GPCR	Engineered	Type of ligand	Ligand name	PDB ID (Resolution Å) [Reference]
A_2A_R (human)	IC3 fusion	Agonist	UK-432097	3QAK (2.71) [101]
		Inverse agonist	ZM241385	3EML (2.6) [98]
	Point mutations	Agonist	Adenosine	2YDO (3.0) [105]
		Agonist	NECA	2YDV (2.6) [105]
		Antagonist	Caffeine	3RFM (3.60) [106]
		Antagonist	XAC	3REY (3.31) [106]
		Inverse agonist	ZM241385	3PWH (3.30) [106]
β_1_AR (turkey)	Point mutations	Agonist	Carmoterol	2Y02 (2.6) [104]
		Agonist	Isoprenaline	2Y03 (2.85] [104]
		Antagonist	Cyanopindolol	2VT4 (2.7) [74], 2YCX (3.25) [159], 2YCY (3.15) [159], 2YCZ (3.65) [159]
		Inverse agonist	Carazolol	2YCW (3.0) [159]
		Partial agonist	Dobutamine	2Y00 (2.5) [104], 2Y01 (2.6) [104]
		Partial agonist	Salbutamol	2Y04 (3.05) [104]
β_2_AR (human)	IC3 fusion	Agonist	BI-167107, nanobody	3P0G (3.5) [96]
		Agonist	FAUC50	3PDS (3.5) [96]
		Antagonist	Alprenolol	3NYA (3.16) [97]
		Inverse agonist	Carazolol	2RH1 (2.4) [18]
		Inverse agonist	Compound #1	3NY9 (2.84) [97]
		Inverse agonist	ICI118551	3NY8 (2.84 [97]
		Inverse agonist	Timolol	3DS4 (2.8) [59]
		Inverse agonist	FAB, not resolved	2R4R (3,4) [96], 2R4S (3.4) [96]
		Inverse agonist	FAB, not resolved	3KJ6 (3.4) [135]
	N-terminal fusion	Agonist	BI-167107, Gαβγ, nanobody	3SN6 (3.2) [15]
CXCR4 (human)	IC3 fusion	Antagonist	CVX15 peptide	3OE0 (2.9) [33]
		Antagonist	Molecule 1t	3ODU (2.5) [33], 3OE6 (3.2) [33], 3OE8 (3.1) [33], 3OE9 (3.1)[33]
D_3_R (human)	IC3 fusion	Antagonist	Eticlopride	3PBL (2.89) [99]
H_1_R (human)	IC3 fusion	Inverse agonist	Doxepin	3RZE (3.1) 36 [100]
Opsin				3CAP (2.9) [89]
			Gα peptide	3DQB (3.2) [91]
Rhodopsin (bovine)		Agonist	All-*trans*-retinal	2G87 (2.6) [139]
		Inverse agonist	11-*cis*-retinal	1F88 (2.8) [17], 1U19 (2.2) [83], 1GZM (2.65) [83], L9H (2.6) [83], 1HZX (2.8) [83], 2I37 (4.0) [83], 3OAX (2.6) [83], 3C9L (2.65) [83]
	Point mutations	Agonist	11-*trans*-retinal, Ga peptide	2X72 (3.0) [122], 3PQR (2.85) [123], 3PXO (3.0) [123]
			9-*cis*-retinal	2PED (2.95) 3385 [85]
				2J4Y (3.4) 3 [83], 3C9M (3.4) [83]
Rhodopsin (squid)		Inverse agonist	11-*cis*-retinal	2ZIY (3.7) [86]

## ABBREVIATIONS

5-HT: = 5-hydroxytryptamine
AFM: = atomic force microscopy
AR: = adrenergic receptor
BRET: = bioluminescence resonance energy transfer
CRD: = cysteine-rich domain
CXCR: = CXC chemokine receptor
D3R: = dopamine receptor D_3_
DFS: = Dynamic Force Spectroscopy
DPPC: = dipalmitoylphospatidylcholine
EC: = extracellular
F-D: = force-distance curves
FRET: = fluorescence resonance energy transfer
GABA: = gamma-aminobutyric acid
GDP: = guanosine diphosphate
GNTI: = guanidinonaltriondole
GPCR: = G protein-coupled receptor
GRK: = GPCR kinase
GTP: = guanosine triphosphate
HBD: = hormone-binding domain
HTS: = high-throughput screening
IC: = intracellular
LRR: = leucine-rich repeat
Mab5: = monoclonal antibody 5
MD: = molecular dynamics
mGlu: = metaboprotic glutamate
MPEP: = 2-methyl-6-(phenylethynyl)-pirydine
NAM: = negative allosteric modulator
OPR: = opioid receptor
PAM: = positive allosteric modulator
SMD: = steered molecular dynamics
SMFS: = Single Molecule Force Spectroscopy
T4L: = T4 lysozyme
TM: = transmembrane region
VFTM: = Venus flytrap module.
